# Supplementary material for: Splice-Junction-Based Mapping of Alternative Isoforms in the Human Proteome
Source: Cell Rep. Author manuscript; Available in PMC 2020 Jan 15. (PMC6961840; doi:10.1016/j.celrep.2019.11.026)
Supplement: 2 [file NIHMS1546469-supplement-2.zip › DF1/PXD006675/RightVentricle/RightVentricle_5_MYBPC3_TDSHEDTGILDFSSLLK.pdf]

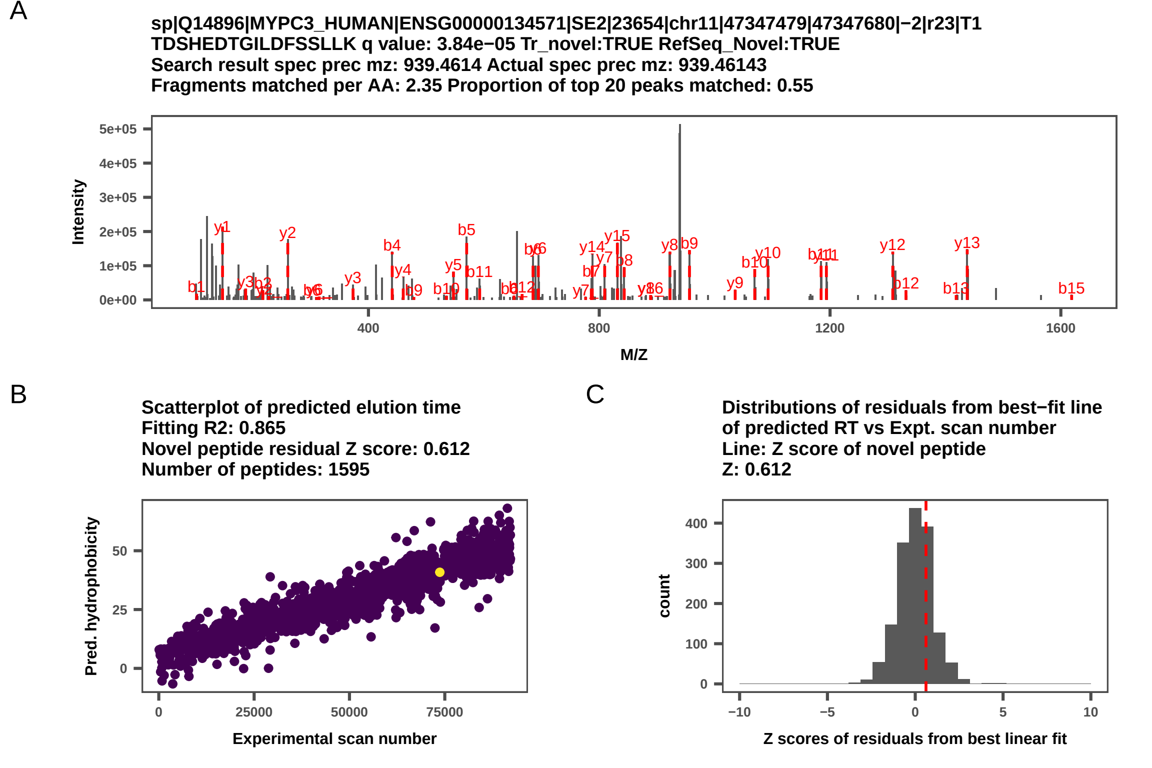

A
sp|Q14896|MYPC3_HUMAN|ENSG00000134571|SE2|23654|chr11|47347479|47347680|−2|r23|T1
TDSHEDTGILDFSSLLK q value: 3.84e−05 Tr_novel:TRUE RefSeq_Novel:TRUE
Search result spec prec mz: 939.4614 Actual spec prec mz: 939.46143
Fragments matched per AA: 2.35 Proportion of top 20 peaks matched: 0.55
Intensity
5e+05
4e+05
3e+05
2e+05
1e+05
0e+00
M/Z
400
800
1200
1600
y1
y2
b4
b5
y5
b11
y4
y3
b9
b1
y14
y15
y7
b8
y8
b9
b10
y10
y9
y12
b12
y13
b13
b15
B
Scatterplot of predicted elution time
Fitting R2: 0.865
Novel peptide residual Z score: 0.612
Number of peptides: 1595
Pred. hydrophobicity
Experimental scan number
0
25
50
0
25000
50000
75000
C
Distributions of residuals from best−fit line
of predicted RT vs Expt. scan number
Line: Z score of novel peptide
Z: 0.612
count
Z scores of residuals from best linear fit
0
100
200
300
400
−10
−5
0
5
10
